# *catena*-Poly[[[diaqua­bis(2-methyl-6-oxo-1,6-dihydro-3,4′-bipyridine-5-carbo­nitrile)copper(II)]-μ-sulfato] tetra­hydrate]

**DOI:** 10.1107/S1600536808038919

**Published:** 2008-11-26

**Authors:** Cao-Yuan Niu, Ai-Min Ning, Chao-Ling Feng, Yu-Li Dang, Chun-Hong Kou

**Affiliations:** a College of Sciences, Henan Agricultural University, Zhengzhou 450002, People’s Republic of China

## Abstract

In the title polymer, {[Cu(SO_4_)(C_12_H_9_N_3_O)_2_(H_2_O)_2_]·4H_2_O}_*n*_, both the metal center and the sulfate anion are located on a twofold axis. The Cu^II^ ion is coordinated by two pyridyl N atoms from two symmetry-related organic ligands, two O atoms from two symmetry-related water mol­ecules, and two O atoms from two symmetry-related sulfate anions, resulting in a distorted octa­hedral geometry. The sulfate anions act as μ_2_-bridges and connect metal ions, forming a one-dimensional chain along the *b* axis. The three-dimensional crystal structure is established through inter­molecular N—H⋯O and O—H⋯O hydrogen bonds involving the organic ligands, sulfate anions, coordinated and uncoordinated water mol­ecules, and through π–π inter­acting 2-pyridone rings, with centroid–centroid separations of *ca* 3.96 Å and tilt angles of *ca* 2.62°.

## Related literature

For background on metal-organic frameworks using sulfate ions as bridging ligands, see: Carlucci *et al.* (2003 ▶); Niu *et al.* (2008 ▶); Xu *et al.* (2003 ▶).
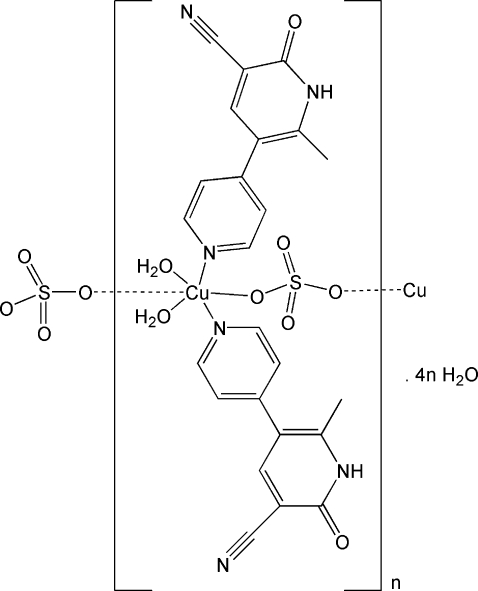


## Experimental

### 

#### Crystal data

[Cu(SO_4_)(C_12_H_9_N_3_O)_2_(H_2_O)_2_]·4H_2_O
*M* *_r_* = 690.14
Orthorhombic, 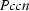

*a* = 21.672 (3) Å
*b* = 6.8533 (8) Å
*c* = 19.860 (3) Å
*V* = 2949.8 (6) Å^3^
*Z* = 4
Mo *K*α radiation
μ = 0.88 mm^−1^
*T* = 291 (2) K
0.32 × 0.23 × 0.22 mm

#### Data collection

Siemens SMART CCD area-detector diffractometer
Absorption correction: multi-scan (*SADABS*; Siemens, 1996 ▶) *T*_min_ = 0.764, *T*_max_ = 0.828
14282 measured reflections
2751 independent reflections
2195 reflections with *I* > 2σ(*I*)
*R*_int_ = 0.034

#### Refinement

*R*[*F* ^2^ > 2σ(*F* ^2^)] = 0.033
*wR*(*F* ^2^) = 0.091
*S* = 1.03
2751 reflections
201 parameters
H-atom parameters constrained
Δρ_max_ = 0.43 e Å^−3^
Δρ_min_ = −0.41 e Å^−3^

### 

Data collection: *SMART* (Siemens, 1996 ▶); cell refinement: *SAINT* (Siemens, 1996 ▶); data reduction: *SAINT*; program(s) used to solve structure: *SHELXL97* (Sheldrick, 2008 ▶); program(s) used to refine structure: *SHELXL97* (Sheldrick, 2008 ▶); molecular graphics: *DIAMOND* (Brandenburg, 2005 ▶); software used to prepare material for publication: *SHELXL97*.

## Supplementary Material

Crystal structure: contains datablocks I, global. DOI: 10.1107/S1600536808038919/bh2204sup1.cif

Structure factors: contains datablocks I. DOI: 10.1107/S1600536808038919/bh2204Isup2.hkl

Additional supplementary materials:  crystallographic information; 3D view; checkCIF report

## Figures and Tables

**Table:** 

Cu1—O4^i^	2.0093 (17)
Cu1—O4	2.0093 (17)
Cu1—N1	2.0197 (19)
Cu1—N1^i^	2.0197 (19)
Cu1—O2	2.3450 (18)
Cu1—O2^i^	2.3450 (18)

**Table:** 

O4^i^—Cu1—O4	90.42 (10)
O4^i^—Cu1—N1	176.94 (7)
O4—Cu1—N1	88.10 (8)
O4^i^—Cu1—N1^i^	88.10 (8)
O4—Cu1—N1^i^	176.94 (8)
N1—Cu1—N1^i^	93.50 (11)
O4^i^—Cu1—O2	86.57 (6)
O4—Cu1—O2	89.37 (7)
N1—Cu1—O2	90.73 (7)
N1^i^—Cu1—O2	93.21 (8)
O4^i^—Cu1—O2^i^	89.37 (7)
O4—Cu1—O2^i^	86.58 (6)
N1—Cu1—O2^i^	93.21 (8)
N1^i^—Cu1—O2^i^	90.73 (7)
O2—Cu1—O2^i^	174.24 (9)

Symmetry code: (i) 
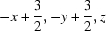
.

**Table 2:** Hydrogen-bond geometry (Å, °)

*D*—H⋯*A*	*D*—H	H⋯*A*	*D*⋯*A*	*D*—H⋯*A*
N2—H2*D*⋯O3^ii^	0.86	1.99	2.847 (3)	173
O4—H2*W*⋯O5	0.82	1.90	2.709 (3)	167
O5—H4*W*⋯O3	0.83	2.10	2.922 (3)	170
O4—H1*W*⋯O3^iii^	0.82	1.93	2.727 (2)	164
O5—H3*W*⋯O6^iv^	0.83	1.94	2.763 (4)	171
O6—H6*W*⋯O1^v^	0.83	2.05	2.737 (4)	139

Symmetry codes: (ii) 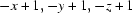; (iii) 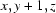; (iv) 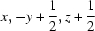; (v) 
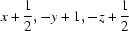
.

**Fig. 1.**
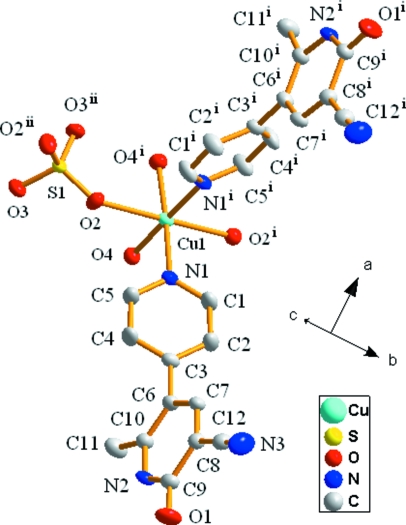
A view of the CuII coordination environment in the polymeric structure of (I), showing the atom-labelling scheme. Displacement ellipsoids are drawn at the 50% probability level. All H atoms and solvent water molecules are omitted for clarity. [Symmetry codes: (i) -x + 3/2, -y + 3/2, z; (ii) -x + 3/2, -y + 1/2, z].

**Fig. 2.**
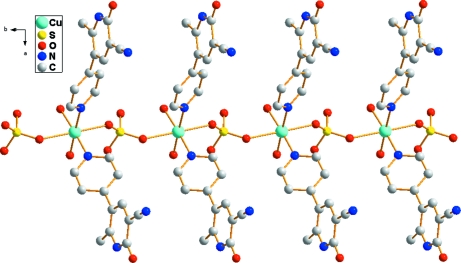
A ball-stick diagram showing the one-dimensional chain. All water molecules and H atoms have been omitted for clarity.

**Fig. 3.**
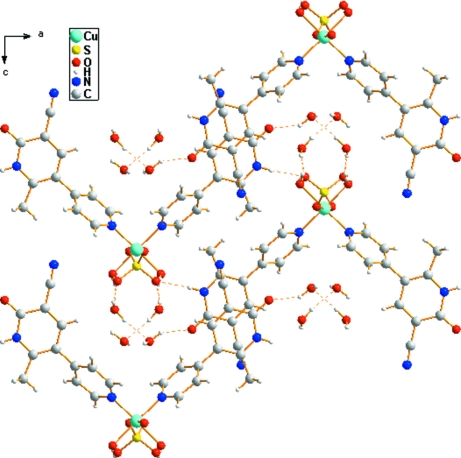
A diagram showing the intermolecular hydrogen bonds indicated by dashed lines.

## supplementary crystallographic information

### Comment

The coordinating modes of sulfate anions can be µ_2_, µ_3_, and µ_4_ bridges that have been used to construct metal-organic frameworks (Carlucci, *et al.*, 2003; Xu, *et al.*, 2003; Niu, *et al.*, 2008).

In the title compound, (I), the central copper ion is coordinated by two N atoms from two symmetry-related organic ligands [N1, N1^i^; symmetry code: (i) -*x* + 3/2, -*y* + 3/2, *z*], two O atoms from two symmetry-related sulfate anions (O2, O2^i^), and two symmetry-related water O atoms (O4, O4^i^), forming a slightly distorted octahedral coordination environment (Fig. 1). The *trans* bond angles around metal centers are in the range 174.24 (9)–176.94 (8)°, close to 180 °, and the *cis* bond angles are in the range 86.58 (6)–93.50 (11) °, close to the right angle (Table 1).

Sulfate anions in the title compound act as µ_2_-bridging ligands to connect copper ions together, forming a one-dimensional chain along *b* axis. The separation of two neighbouring copper atoms in one chain is about 6.85 Å. The organic molecules, 1,6-dihydro-2-methyl-6-oxo-(3,4'-bipyridine)-5-carbonitrile, act as terminal ligands, being coordinated to the copper atoms in chains only through pyridyl N atoms, with the other N and O atoms remaining uncoordinated (Fig. 2). The S1 atom of the sulfate anion is located on a special position of space group *Pccn*, bonding four symmetry-related oxygen atoms [O2, O2^ii^, O3, O3^ii;^ symmetry code: (ii) -*x* + 3/2, -*y* + 1/2, *z*]

There are hydrogen bonds involving organic ligands, sulfate anions, coordinated water molecules, and solvent water molecules. All O atoms of water molecules can either act as donors or as acceptors. Uncoordinating N atoms of pyridone rings only act as donors and sulfate O atoms as acceptors. Neighbouring chains are connected together by these hydrogen bonds (Fig. 3). In addition to these intermolecular hydrogen bonds, there are weak π-π interactions between parallel pyridone rings from two neighbouring chains, with centroid to centroid distances of about 3.96 Å and dihedral angles of about 2.62°.

### Experimental

A solution of CuSO_4_^.^5H_2_O (0.025 g, 0.1 mmol) in CH_3_OH (10 ml) was added to a solution of 1,6-dihydro-2-methyl-6-oxo-(3,4'-bipyridine)-5-carbonitrile (0.021 g, 0.1 mmol) in CH_3_OH (20 ml) under stirring. The mixture was filtered and the resulting solution allowed to evaporate slowly. About 40 days later, blue block single crystals suitable for X-ray analysis were obtained (yield: *ca*. 35%).

### Refinement

H atoms of water molecules were first found in a difference map and refined freely, with *U*_iso_(H) = 1.5*U*_eq_(carrier O). The remaining H atoms were positioned geometrically and refined using a riding model [C—H = 0.93 Å and *U*_iso_(H) = 1.2*U*_eq_(C) for aromatic H atoms; N—H = 0.86 Å and *U*_iso_(H) = 1.2*U*_eq_(N); C—H = 0.96 Å and *U*_iso_(H) = 1.5*U*_eq_(C) for methyl H atoms]. The final difference map had a highest peak at 0.62 Å from atom H6W and a deepest hole at 0.55 Å from atom Cu1, but were otherwise featureless.

### Crystal data

**Table:**

[Cu(SO_4_)(C_12_H_9_N_3_O)_2_(H_2_O)_2_]·4H_2_O	*F*_000_ = 1428
*M**_r_* = 690.14	*D*_x_ = 1.554 Mg m^−^^3^
Orthorhombic, *P**c**c**n*	Mo *K*α radiation λ = 0.71073 Å
Hall symbol: -P 2ab 2ac	Cell parameters from 3682 reflections
*a* = 21.672 (3) Å	θ = 2.3–25.8º
*b* = 6.8533 (8) Å	µ = 0.88 mm^−^^1^
*c* = 19.860 (3) Å	*T* = 291 (2) K
*V* = 2949.8 (6) Å^3^	Block, blue
*Z* = 4	0.32 × 0.23 × 0.22 mm

### Data collection

**Table:**

Siemens SMART CCD area-detector diffractometer	2751 independent reflections
Radiation source: fine-focus sealed tube	2195 reflections with *I* > 2σ(*I*)
Monochromator: graphite	*R*_int_ = 0.034
*T* = 291(2) K	θ_max_ = 25.5º
φ and ω scans	θ_min_ = 2.3º
Absorption correction: multi-scan(SADABS; Siemens, 1996)	*h* = −23→26
*T*_min_ = 0.764, *T*_max_ = 0.828	*k* = −8→8
14282 measured reflections	*l* = −24→24

### Refinement

**Table:**

Refinement on *F*^2^	Secondary atom site location: difference Fourier map
Least-squares matrix: full	Hydrogen site location: inferred from neighbouring sites
*R*[*F*^2^ > 2σ(*F*^2^)] = 0.033	H-atom parameters constrained
*wR*(*F*^2^) = 0.091	*w* = 1/[σ^2^(*F*_o_^2^) + (0.0412*P*)^2^ + 2.9734*P*] where *P* = (*F*_o_^2^ + 2*F*_c_^2^)/3
*S* = 1.03	(Δ/σ)_max_ = 0.001
2751 reflections	Δρ_max_ = 0.43 e Å^−^^3^
201 parameters	Δρ_min_ = −0.41 e Å^−^^3^
Primary atom site location: structure-invariant direct methods	Extinction correction: none

### Fractional atomic coordinates and isotropic or equivalent isotropic displacement parameters (Å^2^)

**Table:**

	*x*	*y*	*z*	*U*_iso_*/*U*_eq_	
Cu1	0.7500	0.7500	0.559997 (19)	0.02153 (13)	
S1	0.7500	0.2500	0.60744 (4)	0.02089 (19)	
O1	0.39746 (9)	0.8422 (3)	0.20721 (9)	0.0447 (5)	
O2	0.73015 (9)	0.4141 (3)	0.56593 (9)	0.0336 (4)	
O3	0.69854 (8)	0.1865 (3)	0.65199 (9)	0.0321 (4)	
O4	0.68578 (8)	0.7952 (2)	0.63128 (9)	0.0291 (4)	
H1W	0.6886	0.9077	0.6453	0.044*	
H2W	0.6925	0.7125	0.6601	0.044*	
O5	0.69067 (10)	0.5382 (3)	0.73479 (10)	0.0464 (5)	
H3W	0.7171	0.5327	0.7654	0.070*	
H4W	0.6888	0.4332	0.7139	0.070*	
O6	0.78398 (17)	0.0151 (9)	0.32780 (19)	0.183 (3)	
H5W	0.7713	0.1316	0.3329	0.275*	
H6W	0.8145	0.0156	0.3023	0.275*	
N1	0.68294 (9)	0.7831 (3)	0.49032 (10)	0.0250 (5)	
N2	0.42947 (9)	0.8330 (3)	0.31661 (10)	0.0282 (5)	
H2D	0.3915	0.8317	0.3292	0.034*	
N3	0.52884 (13)	0.8349 (4)	0.10367 (12)	0.0514 (7)	
C1	0.67866 (13)	0.9364 (4)	0.44882 (13)	0.0357 (7)	
H1	0.7090	1.0323	0.4509	0.043*	
C2	0.63105 (12)	0.9583 (4)	0.40299 (14)	0.0371 (7)	
H2	0.6300	1.0665	0.3747	0.044*	
C3	0.58505 (11)	0.8190 (4)	0.39931 (12)	0.0268 (5)	
C4	0.58919 (13)	0.6623 (4)	0.44327 (14)	0.0392 (7)	
H4A	0.5590	0.5659	0.4430	0.047*	
C5	0.63819 (12)	0.6500 (4)	0.48731 (14)	0.0374 (7)	
H5	0.6401	0.5437	0.5163	0.045*	
C6	0.53443 (11)	0.8312 (4)	0.34882 (12)	0.0260 (5)	
C7	0.54932 (11)	0.8372 (4)	0.27966 (12)	0.0265 (5)	
H7	0.5906	0.8407	0.2670	0.032*	
C8	0.50482 (11)	0.8381 (4)	0.23072 (11)	0.0269 (5)	
C9	0.44027 (11)	0.8380 (4)	0.24792 (12)	0.0280 (6)	
C10	0.47276 (11)	0.8298 (4)	0.36634 (12)	0.0270 (5)	
C11	0.44750 (13)	0.8324 (5)	0.43639 (13)	0.0405 (7)	
H11A	0.4237	0.9490	0.4430	0.061*	
H11B	0.4809	0.8295	0.4681	0.061*	
H11C	0.4216	0.7204	0.4431	0.061*	
C12	0.51933 (12)	0.8364 (4)	0.16012 (13)	0.0318 (6)	

### Atomic displacement parameters (Å^2^)

**Table:**

	*U*^11^	*U*^22^	*U*^33^	*U*^12^	*U*^13^	*U*^23^
Cu1	0.0154 (2)	0.0276 (2)	0.0216 (2)	−0.00008 (18)	0.000	0.000
S1	0.0153 (4)	0.0200 (4)	0.0273 (4)	0.0008 (4)	0.000	0.000
O1	0.0265 (10)	0.0688 (15)	0.0387 (10)	0.0036 (10)	−0.0118 (9)	−0.0038 (10)
O2	0.0356 (10)	0.0244 (10)	0.0408 (10)	−0.0002 (8)	−0.0124 (8)	0.0060 (8)
O3	0.0225 (9)	0.0322 (10)	0.0417 (10)	−0.0012 (8)	0.0090 (8)	−0.0015 (8)
O4	0.0268 (10)	0.0271 (9)	0.0335 (9)	−0.0005 (7)	0.0046 (8)	0.0011 (7)
O5	0.0499 (13)	0.0423 (12)	0.0469 (12)	0.0064 (10)	0.0111 (10)	−0.0005 (9)
O6	0.073 (2)	0.360 (8)	0.116 (3)	−0.085 (4)	−0.016 (2)	0.094 (4)
N1	0.0171 (10)	0.0333 (12)	0.0245 (10)	−0.0016 (9)	−0.0005 (8)	0.0027 (9)
N2	0.0143 (10)	0.0381 (12)	0.0323 (11)	0.0005 (10)	0.0005 (8)	−0.0017 (10)
N3	0.0542 (17)	0.0684 (19)	0.0316 (14)	0.0124 (15)	0.0024 (12)	0.0037 (13)
C1	0.0284 (15)	0.0395 (16)	0.0393 (15)	−0.0127 (13)	−0.0094 (12)	0.0105 (12)
C2	0.0317 (15)	0.0414 (16)	0.0382 (15)	−0.0090 (13)	−0.0098 (12)	0.0167 (12)
C3	0.0186 (12)	0.0360 (14)	0.0260 (12)	0.0003 (11)	−0.0011 (10)	0.0009 (11)
C4	0.0291 (15)	0.0370 (15)	0.0516 (17)	−0.0134 (13)	−0.0144 (13)	0.0133 (14)
C5	0.0294 (15)	0.0379 (16)	0.0451 (15)	−0.0084 (13)	−0.0117 (12)	0.0156 (13)
C6	0.0208 (13)	0.0299 (13)	0.0274 (12)	−0.0020 (11)	−0.0042 (10)	0.0029 (11)
C7	0.0172 (12)	0.0298 (13)	0.0325 (13)	0.0002 (11)	−0.0013 (10)	0.0044 (11)
C8	0.0252 (13)	0.0289 (13)	0.0265 (12)	0.0016 (11)	0.0000 (10)	0.0018 (10)
C9	0.0229 (13)	0.0302 (14)	0.0309 (13)	0.0004 (11)	−0.0040 (11)	−0.0013 (11)
C10	0.0220 (13)	0.0307 (13)	0.0284 (12)	−0.0008 (11)	−0.0019 (10)	0.0005 (11)
C11	0.0299 (15)	0.0608 (19)	0.0309 (14)	−0.0027 (15)	0.0027 (11)	−0.0016 (14)
C12	0.0278 (14)	0.0343 (15)	0.0333 (15)	0.0037 (12)	−0.0023 (11)	0.0031 (12)

### Geometric parameters (Å, °)

**Table:**

Cu1—O4^i^	2.0093 (17)	N2—H2D	0.8600
Cu1—O4	2.0093 (17)	N3—C12	1.140 (3)
Cu1—N1	2.0197 (19)	C1—C2	1.384 (4)
Cu1—N1^i^	2.0197 (19)	C1—H1	0.9300
Cu1—O2	2.3450 (18)	C2—C3	1.382 (4)
Cu1—O2^i^	2.3450 (18)	C2—H2	0.9300
S1—O2	1.4592 (17)	C3—C4	1.387 (4)
S1—O2^ii^	1.4592 (17)	C3—C6	1.489 (3)
S1—O3^ii^	1.4885 (17)	C4—C5	1.378 (4)
S1—O3	1.4885 (17)	C4—H4A	0.9300
O1—C9	1.231 (3)	C5—H5	0.9300
O4—H1W	0.8217	C6—C10	1.381 (3)
O4—H2W	0.8188	C6—C7	1.412 (3)
O5—H3W	0.8349	C7—C8	1.369 (3)
O5—H4W	0.8320	C7—H7	0.9300
O6—H5W	0.8509	C8—C12	1.437 (4)
O6—H6W	0.8326	C8—C9	1.440 (3)
N1—C5	1.333 (3)	C10—C11	1.495 (3)
N1—C1	1.339 (3)	C11—H11A	0.9600
N2—C10	1.363 (3)	C11—H11B	0.9600
N2—C9	1.385 (3)	C11—H11C	0.9600
			
O4^i^—Cu1—O4	90.42 (10)	C2—C1—H1	118.6
O4^i^—Cu1—N1	176.94 (7)	C3—C2—C1	119.9 (2)
O4—Cu1—N1	88.10 (8)	C3—C2—H2	120.1
O4^i^—Cu1—N1^i^	88.10 (8)	C1—C2—H2	120.1
O4—Cu1—N1^i^	176.94 (8)	C2—C3—C4	117.0 (2)
N1—Cu1—N1^i^	93.50 (11)	C2—C3—C6	121.9 (2)
O4^i^—Cu1—O2	86.57 (6)	C4—C3—C6	121.0 (2)
O4—Cu1—O2	89.37 (7)	C5—C4—C3	119.8 (3)
N1—Cu1—O2	90.73 (7)	C5—C4—H4A	120.1
N1^i^—Cu1—O2	93.21 (8)	C3—C4—H4A	120.1
O4^i^—Cu1—O2^i^	89.37 (7)	N1—C5—C4	123.2 (2)
O4—Cu1—O2^i^	86.58 (6)	N1—C5—H5	118.4
N1—Cu1—O2^i^	93.21 (8)	C4—C5—H5	118.4
N1^i^—Cu1—O2^i^	90.73 (7)	C10—C6—C7	117.8 (2)
O2—Cu1—O2^i^	174.24 (9)	C10—C6—C3	122.9 (2)
O2—S1—O2^ii^	111.21 (15)	C7—C6—C3	119.2 (2)
O2—S1—O3^ii^	109.36 (10)	C8—C7—C6	122.0 (2)
O2^ii^—S1—O3^ii^	109.88 (10)	C8—C7—H7	119.0
O2—S1—O3	109.88 (10)	C6—C7—H7	119.0
O2^ii^—S1—O3	109.37 (10)	C7—C8—C12	122.6 (2)
O3^ii^—S1—O3	107.06 (15)	C7—C8—C9	121.1 (2)
S1—O2—Cu1	136.96 (10)	C12—C8—C9	116.4 (2)
Cu1—O4—H1W	109.4	O1—C9—N2	121.3 (2)
Cu1—O4—H2W	105.3	O1—C9—C8	125.2 (2)
H1W—O4—H2W	113.6	N2—C9—C8	113.5 (2)
H3W—O5—H4W	110.9	N2—C10—C6	118.9 (2)
H5W—O6—H6W	108.9	N2—C10—C11	115.0 (2)
C5—N1—C1	117.3 (2)	C6—C10—C11	126.1 (2)
C5—N1—Cu1	118.51 (17)	C10—C11—H11A	109.5
C1—N1—Cu1	124.06 (17)	C10—C11—H11B	109.5
C10—N2—C9	126.7 (2)	H11A—C11—H11B	109.5
C10—N2—H2D	116.6	C10—C11—H11C	109.5
C9—N2—H2D	116.6	H11A—C11—H11C	109.5
N1—C1—C2	122.8 (2)	H11B—C11—H11C	109.5
N1—C1—H1	118.6	N3—C12—C8	177.8 (3)
			
O2^ii^—S1—O2—Cu1	−130.08 (19)	Cu1—N1—C5—C4	177.7 (2)
O3^ii^—S1—O2—Cu1	−8.6 (2)	C3—C4—C5—N1	0.1 (5)
O3—S1—O2—Cu1	108.69 (16)	C2—C3—C6—C10	−124.4 (3)
O4^i^—Cu1—O2—S1	6.07 (17)	C4—C3—C6—C10	58.0 (4)
O4—Cu1—O2—S1	−84.39 (17)	C2—C3—C6—C7	58.1 (4)
N1—Cu1—O2—S1	−172.48 (17)	C4—C3—C6—C7	−119.5 (3)
N1^i^—Cu1—O2—S1	93.97 (17)	C10—C6—C7—C8	−1.2 (4)
O4—Cu1—N1—C5	−66.4 (2)	C3—C6—C7—C8	176.5 (3)
N1^i^—Cu1—N1—C5	116.2 (2)	C6—C7—C8—C12	−177.5 (2)
O2—Cu1—N1—C5	22.9 (2)	C6—C7—C8—C9	1.6 (4)
O2^i^—Cu1—N1—C5	−152.9 (2)	C10—N2—C9—O1	−179.4 (3)
O4—Cu1—N1—C1	110.0 (2)	C10—N2—C9—C8	0.5 (4)
N1^i^—Cu1—N1—C1	−67.4 (2)	C7—C8—C9—O1	178.7 (3)
O2—Cu1—N1—C1	−160.6 (2)	C12—C8—C9—O1	−2.2 (4)
O2^i^—Cu1—N1—C1	23.5 (2)	C7—C8—C9—N2	−1.2 (4)
C5—N1—C1—C2	−1.4 (4)	C12—C8—C9—N2	177.9 (2)
Cu1—N1—C1—C2	−177.9 (2)	C9—N2—C10—C6	−0.2 (4)
N1—C1—C2—C3	0.6 (5)	C9—N2—C10—C11	177.7 (3)
C1—C2—C3—C4	0.5 (4)	C7—C6—C10—N2	0.4 (4)
C1—C2—C3—C6	−177.2 (3)	C3—C6—C10—N2	−177.2 (2)
C2—C3—C4—C5	−0.8 (4)	C7—C6—C10—C11	−177.2 (3)
C6—C3—C4—C5	176.9 (3)	C3—C6—C10—C11	5.2 (5)
C1—N1—C5—C4	1.0 (4)		

Symmetry codes: (i) −*x*+3/2, −*y*+3/2, *z*; (ii) −*x*+3/2, −*y*+1/2, *z*.

### Hydrogen-bond geometry (Å, °)

**Table:**

*D*—H···*A*	*D*—H	H···*A*	*D*···*A*	*D*—H···*A*
N2—H2D···O3^iii^	0.86	1.99	2.847 (3)	173
O4—H2W···O5	0.82	1.90	2.709 (3)	167
O5—H4W···O3	0.83	2.10	2.922 (3)	170
O4—H1W···O3^iv^	0.82	1.93	2.727 (2)	164
O5—H3W···O6^v^	0.83	1.94	2.763 (4)	171
O6—H6W···O1^vi^	0.83	2.05	2.737 (4)	139

Symmetry codes: (iii) −*x*+1, −*y*+1, −*z*+1; (iv) *x*, *y*+1, *z*; (v) *x*, −*y*+1/2, *z*+1/2; (vi) *x*+1/2, −*y*+1, −*z*+1/2.
